# Gastrointestinal Tracking and Gastric Emptying of Coated Capsules in Rats with or without Sedation Using CT imaging

**DOI:** 10.3390/pharmaceutics12010081

**Published:** 2020-01-19

**Authors:** Noemí Gómez-Lado, Iria Seoane-Viaño, Silvia Matiz, Christine M. Madla, Vipul Yadav, Pablo Aguiar, Abdul W. Basit, Alvaro Goyanes

**Affiliations:** 1 Nuclear Medicine Department and Molecular Imaging Group, University Clinical Hospital (CHUS) and Health Research Institute of Santiago de Compostela (IDIS), 15706 A Coruña, Spain; noemi.gomez@usc.es; 2 Department of Pharmacology, Pharmacy and Pharmaceutical Technology, Faculty of Pharmacy, University of Santiago de Compostela (USC), Campus Vida, 15782 Santiago de Compostela, Spain; iriasv92@gmail.com; 3 Intract Pharma, Royal College St, London NW1 0NH, UK; silvia.matiz@intractpharma.com (S.M.); vipul.yadav@intractpharma.com (V.Y.); 4 Department of Pharmaceutics, UCL School of Pharmacy, University College London, 29-39 Brunswick Square, London WC1N 1AX, UK; christine.madla.16@ucl.ac.uk; 5 FabRx Ltd., 3 Romney Road, Ashford TN24 0RW, UK; 6 Departamento de Farmacología, Farmacia y Tecnología Farmacéutica, I + D Farma Group (GI-1645), Universidade de Santiago de Compostela, 15782 Santiago de Compostela, Spain

**Keywords:** medical imaging, computed tomography, anesthesia, capsule size, gastric emptying, rodents, gastrointestinal tract, oral drug delivery, drug absorption

## Abstract

Following oral administration, gastric emptying is often a rate-limiting step in the absorption of drugs and is dependent on both physiological and pharmaceutical factors. To guide translation into humans, small animal imaging during pre-clinical studies has been increasingly used to localise the gastrointestinal transit of solid dosage forms. In contrast to humans, however, anaesthesia is usually required for effective imaging in animals which may have unintended effects on intestinal physiology. This study evaluated the effect of anaesthesia and capsule size on the gastric emptying rate of coated capsules in rats. Computed tomography (CT) imaging was used to track and locate the capsules through the gastrointestinal tract. Two commercial gelatine mini-capsules (size 9 and 9h) were filled with barium sulphate (contrast agent) and coated using Eudragit L. Under the effect of anaesthesia, none of the capsules emptied from the stomach. In non-anaesthetised rats, most of the size 9 capsules did not empty from the stomach, whereas the majority of the smaller size 9h capsules successfully emptied from the stomach and moved into the intestine. This study demonstrates that even with capsules designed to empty from the stomach in rats, the gastric emptying of such solid oral dosage forms is not guaranteed. In addition, the use of anaesthesia was found to abolish gastric emptying of both capsule sizes. The work herein further highlights the utility of CT imaging for the effective visualisation and location of solid dosage forms in the intestinal tract of rats without the use of anaesthesia.

## 1. Introduction

The use of animals in biomedical and pharmaceutical research remains essential to understand the mechanisms underlying disease pathology, as well as to contribute to the discovery of improved methods to prevent, diagnose and treat them [1]. In drug development, animal testing has provided an important tool to guide the translation of formulations from pre-clinical studies to first-in-human (FIH) clinical trials. Among the different animals used in preclinical research, rodents make up approximately 95% of all laboratory animals [2]. Specifically, rats are one of the most widely used small animal models for the assessment of new pharmaceutical entities during pre-clinical studies [3,4,5,6,7,8,9].

The oral route is the most preferred and convenient way to administer drugs which can either be formulated to act locally in the gastrointestinal (GI) tract or be absorbed to achieve a systemic effect [10]. Hard shell capsules are commonly used for oral administration of drugs. Uncoated gelatine capsules, however, rapidly disintegrate and immediately release the drug in the stomach [11]. If a modified release of the drug in lower regions of the GI tract is pursued, a coating is generally applied on the capsule surface [12,13].

To facilitate the oral administration of solid dosage forms in rats, anaesthesia is often used to prevent internal injury [14]. The use of sedative agents, however, has been shown to influence the rate of gastric emptying in animals. When the effect of several anaesthetic agents on rat duodenum motility was investigated, all of the anaesthetic agents studied disrupted GI transit [15]. In dogs, a significant decrease in gastric myoelectric and motor activity was reported for 6 h following anaesthesia with isoflurane, and in the gastric motility index for 18 h post-anaesthesia [16]. In rats, even a brief exposure to isoflurane anaesthesia decreased GI motility which did not return to normal until 2 h later [17].

Data in the literature on the gastric emptying of solid oral dosage forms in rats is contradictory. A number of studies have reported the failure of capsules to empty from the stomach [18,19], albeit other investigations have demonstrated the transit of solid oral dosage forms through the GI tract [13,20,21]. These contradictory findings are difficult to interpret and may be related to a multitude of factors.

As such, to better understand intestinal transit, non-invasive imaging techniques have been employed to visualise drug delivery systems along the GI tract [20,21,22,23,24]. Techniques such as magnetic resonance imaging (MRI) [25], single-photon emission computed tomography (SPECT) [13] and X-ray imaging [26] have been applied to track the intestinal movement of solid dosage forms following oral administration. For instance, the relationship between the size of the formulations and its gastric emptying in rats has been investigated using microcapsules radiolabelled with ^99m^Tc-DTPA. Through the use of scintigraphy planar imaging, it was reported that the cut-off emptying size in rats (body weight 250–300 g) was between 1.5 and 2 mm [27]. Another study using X-ray planar imaging found that commercial size 9 mini-capsules (7.18 mm length × 2.64 mm diameter) were retained in the stomach. However, if the capsules were cut in length to 3.5 mm, they emptied from the stomach [28]. Among other imaging techniques, computed tomography (CT) enables the direct 3D imaging and clear differentiation of soft tissue structures. In combination with positron emission tomography (PET), CT was used to investigate the in vivo behaviour of 3D printed capsules of different compositions in the GI tract of rats [18].

The aim of this study was to evaluate the influence of (i) capsule size and (ii) anaesthesia on the gastric emptying of size 9 and 9h capsules which are specifically designed for oral administration to rats. Computed tomography (CT) was used to visualise the coated capsules filled with the contrast agent, barium sulphate, through the GI tract.

## 2. Material and Methods

### 2.1. Materials

Size 9 (8.4 mm length × 2.7 mm diameter) and 9h (5.1 mm length × 2.7 mm diameter) gelatine capsules were purchased from Torpac (Fairfield, NJ, USA). Barium sulphate, reagent grade 99%, was obtained from Honeywell (Bracknell, UK). α-Lactose monohydrate, talc and triethyl citrate, 99% (TEC) were obtained from Sigma-Aldrich (Gillingham, UK). Eudragit L100 (Eudragit L, a pH-sensitive polymer that dissolves above pH 6) was obtained from Evonik (Darmstadt, Germany).

### 2.2. Methods

#### 2.2.1. Capsule Filing

Capsule sizes 9 and 9h were filled with 7.5 mg of barium sulphate. The appropriate mass of lactose was used as a filler to keep the weight of both capsule types consistent (25 mg) (Table 1).

#### 2.2.2. Capsule Coating

The loaded capsules were coated with Eudragit L to avoid disintegration in the stomach. The coating solution was prepared by adding Eudragit L into a mixture of isopropanol (97% based on solvent weight) and water while stirring with a magnetic stirrer plate. Talc (50% based on polymer weight) was added as an anti-tacking agent in a similar manner until a homogenous dispersion was obtained. TEC (10% based on polymer weight) was then added to the dispersion. The total solid content of the final dispersion was 10% *w*/*w*. The capsules were coated using a bottom spray fluidised Tablet and Pellet Mini Coater (Caleva, Sturminster Newton, UK) to a coating thickness of 8 mg/cm^2^ which was selected to provide gastro-resistance. The coating conditions were as follows; Inlet air temperature 40 °C; outlet air temperature 30 °C; fan capacity at setting 15 (equivalent to air flow 150 m^3^/h); atomising pressure of 0.2 bar and a spray rate of 1.0 mL/min.

#### 2.2.3. In Vivo Study Design

This study was carried out on male Sprague-Dawley rats with an average weight of 350 g supplied by the animal facility at the University of Santiago Compostela (USC). The animals were kept in individual cages with free access to food and water in a room under controlled temperature (22 ± 1 °C) and humidity (60 ± 5%) conditions and with day-night cycles regulated by artificial light (12/12 h). During the experiments, animals were kept in individual cages with a platform (to avoid the ingestion of faeces) and were fasted for 12 h before capsule administration until 4 h post-administration.

All animal experiments complied with the Animal Research: Reporting of In Vivo Experiments (ARRIVE) guidelines [29] and were carried out in accordance with the EU Directive 2010/63/EU for animal experiments, being approved by the Galician Network Committee for Ethics Research (IDIS/2007-17, 20-07-2017). The animals were divided in four groups of six rats (Table 2). At the beginning of the study for the dosing of the formulations, the capsules (size 9 or 9h) were introduced directly into the stomach of the rats using a Torpac dosing device for rodents. The animals in the anaesthesia group were placed in a gas chamber containing 3% isoflurane in oxygen until they were unconscious. No anaesthesia was administered at any point to the animals in groups 2 and 4.

#### 2.2.4. CT Acquisition

Before the acquisition of the CT images, animals in the non-anaesthesia group were restrained using an acrylic tube. Animals in the anaesthesia group were prepared as outlined in Section 2.2.3.

CT scans were performed to study the distribution of the capsules in the GI tract before capsule administration (basal condition) and 2 min, 1, 2, 3, 4, 5, 6 and 24 h post-administration. CT images were acquired using an Albira PET/CT Preclinical Imaging System. The CT subsystem consists of a microfocus X-ray tube of 50 kVp and a CsI scintillator 2D pixilated flat panel detector that can generate images around 90 µm with a FOV of 7 cm. A CT static acquisition was performed, consisting of 15 min CT scan focused on the abdominal region of each animal. Once the study finished, the animals were returned to their cages. All images were analysed visually using AMIDE software to study the location of the capsule at each time point post-administration.

## 3. Results and Discussion

**Figure 1:**
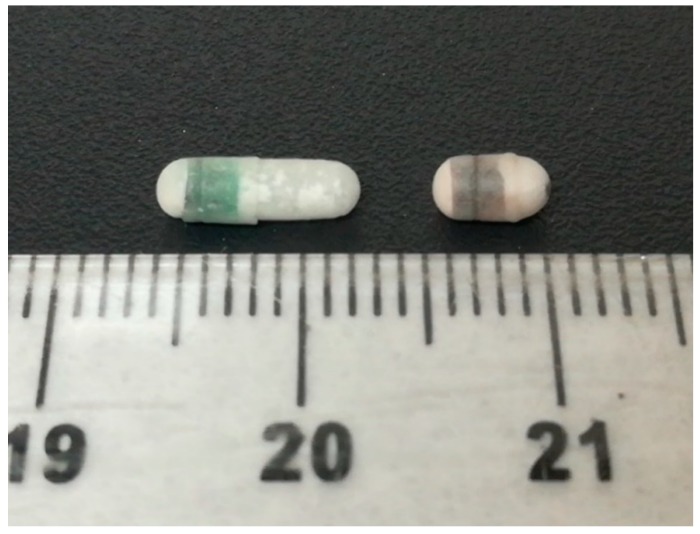
Picture of the coated capsules, size 9 (left) and size 9h (right).

In this study, two different capsule sizes (size 9 and 9h) were filled with barium sulphate which was used as a contrast agent and coated with the polymer Eudragit L (Figure 1).

In order to assess the influence of anaesthesia and capsule size on the gastric emptying of the capsules, the formulations were administered into the stomach of fasted rats and their behaviour in vivo within the GI tract was evaluated by CT imaging.

**Figure 2:**
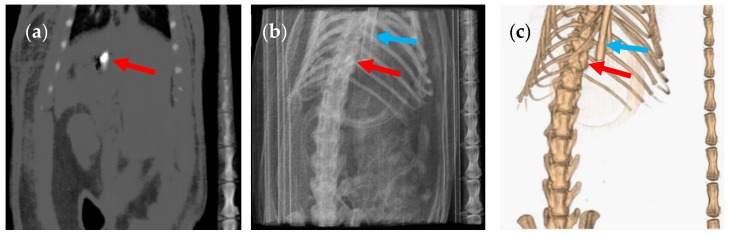
(**a**) Coronal computed tomography (CT) slice of the size 9 capsule in the stomach of the rat, (**b**) planar CT projection and (**c**) three-dimensional rendered CT image of the same animal. The capsule is indicated by a red arrow and the breathing sensor by a blue arrow. Image taken under anaesthesia.

**Figure 3:**
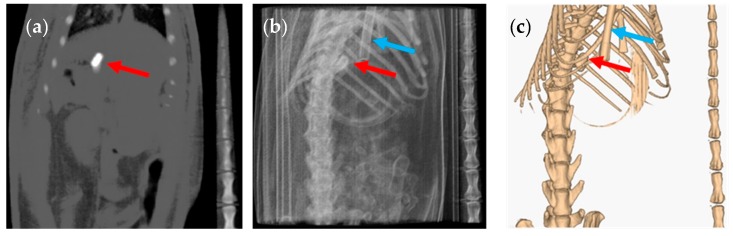
(**a**) Coronal CT slice of the size 9h capsule in the stomach of the rat, (**b**) planar CT projection and (**c**) three-dimensional rendered CT image of the same animal. The capsule is indicated by a red arrow and the breathing sensor by a blue arrow. Image taken under anaesthesia.

The size 9 and 9h capsules were given to two groups of six rats under anaesthesia for the capsule administration and during image acquisition. Both 9 and 9h capsules containing barium sulphate could be clearly seen as a white ellipsoid on the grey colour of the two-dimensional coronal CT slice (Figure 2 and Figure 3 show capsule sizes 9 and 9h respectively). The three-dimensional (3D) CT image of the same animal determined the relative position and location of the capsule (red arrow) in the body of the rat. A breathing sensor that is used to evaluate rat conditions during the test when using anaesthesia is also visible in the 3D images (blue arrow).

**Figure 4:**
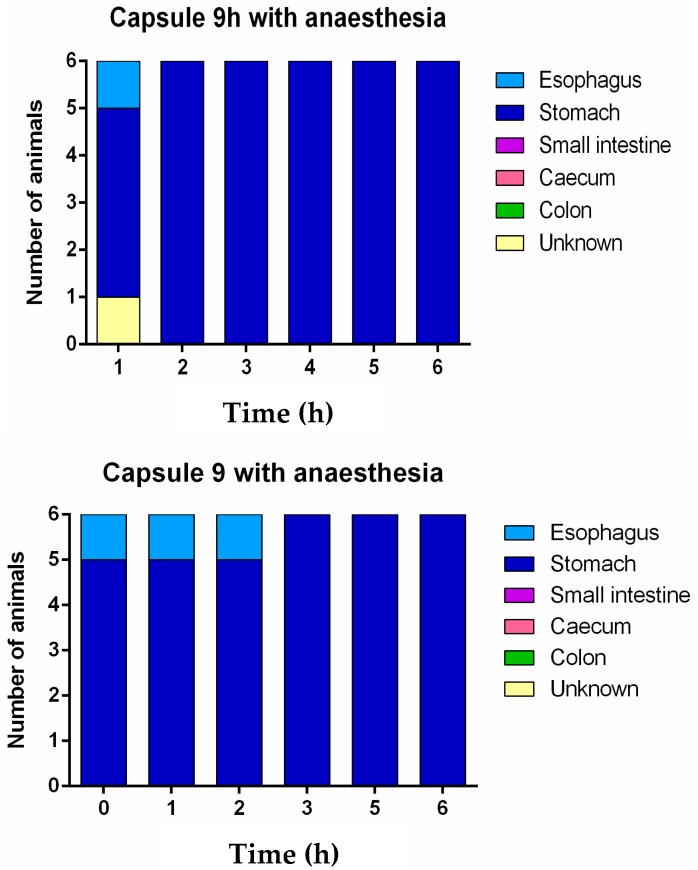
Graphical representation of the location of the capsules at different times under anaesthesia. Unknown denotes the uncertain location of the capsules in the gastrointestinal tract due to the capsule or animal moving during CT image acquisition at the time point.

CT images taken after the administration of the capsules showed that the capsules did not pass through the pylorus to the small intestine. All capsules were retained in the stomach until their disintegration (Figure 4, Supplementary Tables S1 and S2). These results were unexpected as although size 9 and 9h capsules are designed for lower GI tract drug delivery in rats from 150–200 g [30], gastric emptying was not achieved in the rats of approximately 350 g used in the present study. The lack of gastric emptying may be explained by several factors including the fed or fasted state of the animal, stress levels or the influence of anaesthesia.

**Figure 5:**
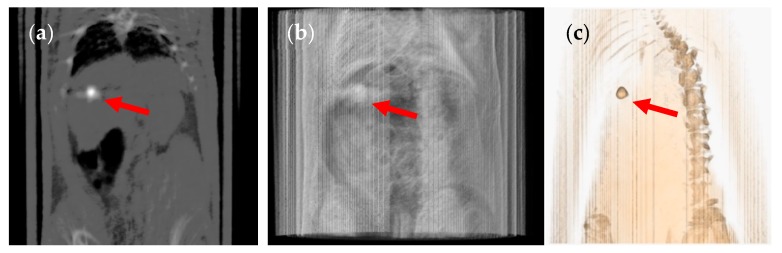
(**a**) Coronal CT slice of the size 9 capsule in the stomach of the rat, (**b**) planar CT projection and (**c**) three-dimensional rendered CT image of the same animal. The capsule is indicated by a red arrow. Image taken without anaesthesia.

The use of anaesthesia is a common practice in animal studies, especially when it is necessary for the animal to remain still during image acquisition. In order to elucidate whether anaesthesia was the reason for the lack of gastric emptying, we performed a second experiment with two different groups of six animals, under the same conditions as the previous experiment, but in the absence of anaesthesia. To do so, we developed an ergonomic cylindrical device to restrain and immobilise the rats during the CT scans. The cylinder was built with transparent acrylic material with two closed ends containing holes for air circulation, providing a proper restraint with comfort and safety. In the absence of anaesthetic conditions, the animals do not remain completely still. As such, the acquired images show lower resolution than those obtained under anaesthesia (Figure 5).

**Figure 6:**
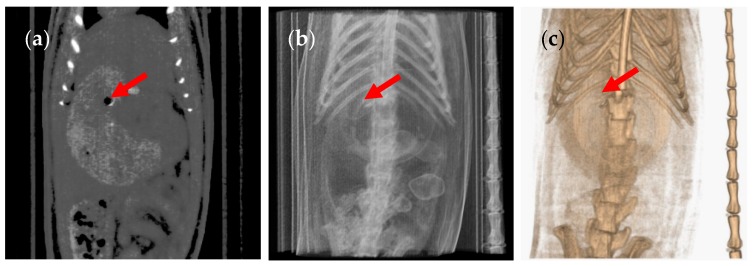
(**a**) Coronal CT slice of an animal with a broken size 9 capsule in the stomach 6 h after administration. The white trail of spots left by the broken capsule is signalled by a red arrow. (**b**) Planar CT projection and (**c**) three-dimensional rendered CT image of the same animal. This animal did not receive anaesthesia.

**Figure 7:**
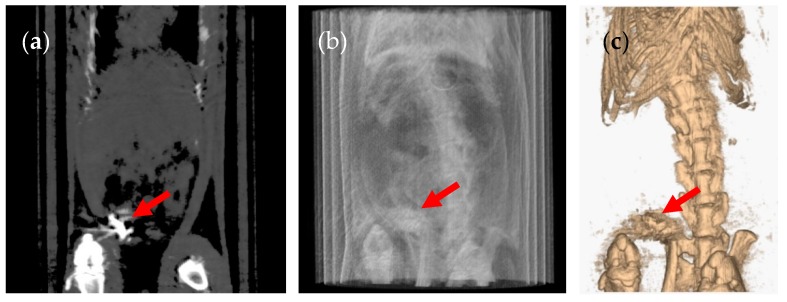
(**a**) Coronal CT slice of an animal with a broken size 9h capsule in the caecum 3 h after administration. The white trail of spots left by the broken capsule is signalled by a red arrow. (**b**) Planar CT projection and (**c**) three-dimensional rendered CT image of the same animal. This animal did not receive anaesthesia.

**Figure 8:**
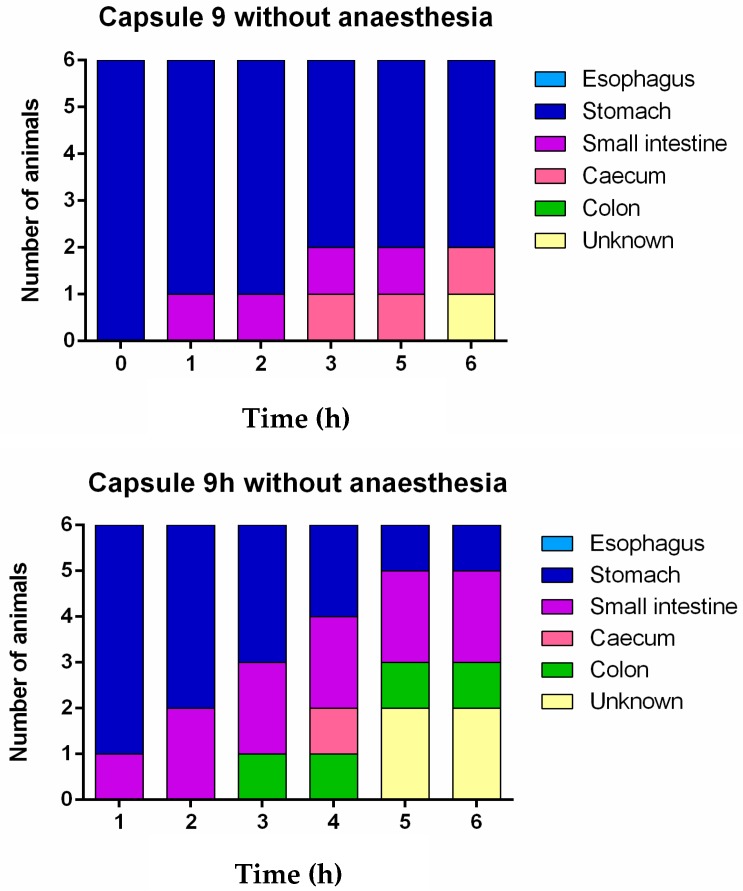
Graphical representation of the location of the capsules at different times without anaesthesia. Unknown denotes the uncertain location of the capsules in the gastrointestinal tract due to the capsule or animal moving during CT image acquisition at the time points.

The CT images showed that in some rats, the capsules successfully emptied the stomach and disintegrated in different regions of the GI tract. The disintegrated capsule can be seen as a white trail of spots (Figure 6 and Figure 7). Figure 8 outlines the location of size 9 and 9h capsules in the GI tract of the rats without anaesthesia. In two of the six animals in the size 9 group, the capsule could be visualised in the intestine 3 h following oral administration. In the remaining animals, the capsules were retained in the stomach until their disintegration. On the other hand, in three of six animals in the size 9h group, the capsules reached the intestine 3 h after administration. Only one animal with a capsule retained in its stomach was identified 5 h following oral administration (Supplementary Tables S3 and S4).

These results confirm the negative influence of anaesthesia on gastric motility which consequently retard the gastric emptying of solid dosage forms in rats. Moreover, the study identified that size 9h capsules (5.1 mm length) more easily pass through the pylorus when compared with size 9 (8.4 mm length) capsules in the absence of anaesthesia.

Some studies have used imaging techniques including SPECT [13] and X-ray [24] to follow dosage forms through the GI tract. However, a number of limitations arise with such techniques as the 2D projection images generated can be poorer in quality with respect to resolution. In addition, it is often difficult to accurately locate the capsule, especially if the capsule is superimposed with organs or bones. On the other hand, CT and MRI, which provide 3D images of complex structures in the form of coronal cuts and 3D rendering with good spatial resolution, were also used for the in vivo visualization of capsules within the GI tract [19,31].

In particular, CT imaging uses special X-ray equipment to generate cross-sectional images of the body [32] in relatively short scan times. Unlike X-ray imaging, CT does not require the use of oral contrast to highlight the digestive organs [28], thus avoiding a time-consuming step since the oral contrast must be administrated before performing the scans and the appropriate amount of contrast has to be optimised to achieve the best possible scan images.

For instance, in a study that used μCT imaging to investigate the gastro-resistance properties of coated capsules [19], gastric emptying was not observed even 5 h after administration, whereas another study that used coated size 9 capsules found that gastric emptying occurred between 2 and 8 h after administration [20]. Furthermore, in another study that used μCT imaging to evaluate the viability of small 3D printed capsular devices, none of the formulations emptied from the stomach, although they were only 3.2 mm in length [18].

However, none of the aforementioned studies considered the potential effects of anaesthesia on gastric motility and did not compare the gastric emptying rates of two commercial capsule sizes (9 and 9h) in rodents. The study herein is of particular relevance for drug formulations to be released in the intestine from coated capsules; if the dosage form has not emptied from the stomach and breaks before reaching the intestine, the results could be interpreted that the drug—or even the study—has failed, even if that is not the problem.

In this work, CT imaging was used to locate, track and evaluate the influence of i) two different capsule sizes and ii) the effect of anaesthesia on gastric emptying. Overall, this study has demonstrated that even with capsules adapted to empty from the stomach in rats, the gastric emptying of such solid oral dosage forms was not guaranteed. The administration of anaesthesia, even for a short period during the experiment—and to a lesser extent the size of the capsules—have been identified as the main contributing factors that negatively influence gastric emptying. In addition, the use of non-invasive CT imaging can drastically reduce the number of animals needed for in vivo experiments and complies with the replacement, reduction and refinement principle.

## 4. Conclusions

The use of anaesthesia clearly retards gastric emptying of capsules in rats independent of capsule size. In non-anaesthetised rats, most of the size 9 capsules did not exit the stomach, whereas the majority of the smaller size 9h capsules successfully emptied from the stomach and moved into the intestine. In comparison to 2D imaging and invasive techniques, CT offers significant benefits in tracking the intestinal movement of solid dosage forms in rats, even in the absence of anaesthesia. The results of this study help to better understand the performance of coated formulations in rats and may explain the failure of this type of formulation to empty from the stomach in previous studies.

## Figures and Tables

**Table 1:** Characteristics of the capsules.

Capsule Size	9	9h
Desired weight of the coated formulation (mg)	25	25
Average weight empty capsule (body + cap) (mg)	10.28	6.38
Surface area locked capsule (cm^2^)	0.69	0.43
Coating thickness (mg/cm^2^)	8	8
Coating per capsule (mg)	5.52	3.44
Barium sulphate (mg)	7.5	7.5
Required amount of lactose (mg)	1.7	7.68

**Table 2:** Groups of study design.

Group Number	Capsule Size	Anaesthesia
1	9	Yes
2	9	No
3	9h	Yes
4	9h	No

## Supplementary Materials

The following are available online at https://www.mdpi.com/1999-4923/12/1/81/s1, Table S1: Location of size 9 capsule with anaesthesia (isoflurane) post-administration, Table S2: Location of size 9h capsule with anaesthesia (isoflurane) post-administration, Table S3: Location of size 9 capsule without anaesthesia post-administration, Table S4: Location of size 9h capsule without anaesthesia post-administration.
