# Supplementary material for: Gastrointestinal Tracking and Gastric Emptying of Coated Capsules in Rats with or without Sedation Using CT imaging
Source: Pharmaceutics. 2020 Jan 19;12(1):81. doi: 10.3390/pharmaceutics12010081 (PMC7023106; doi:10.3390/pharmaceutics12010081)
Supplement: Supplementary file 1 [file pharmaceutics-12-00081-s001.pdf]

pharmaceutics

MDPI

Article

Supplementary Materials: Gastrointestinal Tracking and Gastric Emptying of Coated Capsules in Rats with or without Sedation using CT imaging

Noemí Gómez-Lado [1], Iria Seoane-Viaño [2], Silvia Matiz [3], Christine M. Madla [4], Vipul Yadav [3], Pablo Aguiar [1,*], Abdul W. Basit [4,5,*] and Alvaro Goyanes [5,6,*]

Table S1. Location of size 9 capsule with anaesthesia (isoflurane) post-administration.

Animal	Post administration	1 h	2 h	3 h	5 h	6 h
1	Esophagus	Esophagus	Esophagus	Stomach	Stomach	Stomach
2	Stomach	Stomach	Stomach	Stomach	Stomach	Stomach
3	Stomach	Stomach	Stomach	Stomach	Stomach	Stomach
4	Stomach	Stomach	Stomach	Stomach	Stomach	Stomach
5	Stomach	Stomach	Stomach	Stomach	Stomach	Stomach
6	Stomach	Stomach	Stomach	Stomach	Stomach	Stomach

Table S2. Location of size 9h capsule with anaesthesia (isoflurane) after post-administration.

Animal	Post administration	1 h	2 h	3 h	5 h	6 h
1	Stomach	Stomach	Stomach	Stomach	Stomach	Stomach
2	Stomach	Stomach	Stomach	Stomach	Stomach	Stomach
3	Stomach	Stomach	Stomach	Stomach	Stomach	Stomach
4	Unknown	Stomach	Stomach	Stomach	Stomach	Stomach
5	Stomach	Stomach	Stomach	Stomach	Stomach	Stomach
6	Esophagus	Stomach	Stomach	Stomach	Stomach	Stomach

Table S3. Location of size 9 capsule without anaesthesia post-administration.

Animal	Post administration	1 h	2 h	3 h	5 h	6 h
1	Stomach	Stomach	Stomach	Stomach	Stomach	Stomach
2	Stomach	Stomach	Stomach	Intestine	Intestine	Unknown
3	Stomach	Stomach	Stomach	Stomach	Stomach	Stomach
4	Stomach	Stomach	Stomach	Stomach	Stomach	Stomach
5	Stomach	Stomach	Stomach	Stomach	Stomach	Stomach
6	Stomach	Intestine	Intestine	Caecum	Caecum	Caecum

Table S4. Location of size 9h capsule without anaesthesia post-administration.

Animal	Post administration	1 h	2 h	3 h	5 h	6 h
1	Stomach	Stomach	Stomach	Stomach	Intestine	Intestine
2	Intestine	Intestine	Colon	Colon	Colon	Unknown
3	Stomach	Intestine	Intestine	Caecum	Unknown	Unknown
4	Stomach	Stomach	Intestine	Intestine	Intestine	Intestine
5	Stomach	Stomach	Stomach	Stomach	Stomach	Stomach
6	Stomach	Stomach	Stomach	Intestine	Unknown	Colon
